# Outcome of endovascular treatment within and beyond 6 h without perfusion software

**DOI:** 10.1038/s41598-021-84857-8

**Published:** 2021-03-05

**Authors:** Zhen Jing, Hao Li, Shengming Huang, Min Guan, Yongxin Li, Kui Lu, Jianzhou Wu, Wangtao Zhong, Li’an Huang

**Affiliations:** 1 Department of Neurology, Clinical Neuroscience Institute, The First Affiliated Hospital of Jinan University, Guangzhou, China; 2 Department of Neurology, Maoming People’s Hospital, Maoming, China; 3 Department of Neurology, Shunde Hospital of Southern Medical University, Foshan, China; 4 Department of Neurology, Zhongshan People’s Hospital, Zhongshan, China; 5 Department of Neurology, Yunfu People’s Hospital, Yunfu, China; 6 Department of Neurology, Affiliated Hospital of Guangdong Medical University, Zhanjiang, China

**Keywords:** Cerebrovascular disorders, Stroke

## Abstract

Endovascular treatment (EVT) has been accepted as the standard of care for patients with acute ischemic stroke. The aim of the present study was to compare clinical outcomes of patients who received EVT within and beyond 6 h from symptom onset to groin puncture without perfusion software in Guangdong district, China. Between March 2017 and May 2018, acute ischemic stroke patients who received EVT from 6 comprehensive stroke centers, were enrolled into the registry study. In this subgroup study, we included all patients who had acute proximal large vessel occlusion in the anterior circulation. The demographic, clinical and neuroimaging data were collected from each center. A total of 192 patients were included in this subgroup study. They were divided into two groups: group A (*n* = 125), within 6 h; group B (*n* = 67), 6–24 h from symptom onset to groin puncture. There were no substantial differences between these two groups in terms of 90 days favorable outcome (modified Rankin scale [mRS] ≤ 2, *P* = 0.051) and mortality (*P* = 0.083), and the risk of symptomatic intracranial hemorrhage at 24 h (*P* = 0.425). The NIHSS (median 16, IQR12-20, group A; median 12, IQR8-18, group B; *P* = 0.009) and ASPECTS (median 10, IQR8-10, group A; median 9, IQR8-10, group B; *P* = 0.034) at baseline were higher in group A. The anesthesia method (general anesthesia, 21.3%, group A vs. 1.5% group B, *P* = 0.001) were also statistically different between the two groups. The NIHSS and ASPECTS were higher, and general anesthesia was also more widely used in group A. Clinical outcomes were not significantly different within 6 h versus 6–24 h from symptom onset to groin puncture in this real world study.

## Introduction

Endovascular treatment (EVT) has been proven to be highly effective in patients with acute ischemic stroke caused by proximal intracranial artery occlusion in the anterior circulation^1–6^. Therefore, this treatment became the standard of care in 2015^7^. A meta-analysis of data from the first 5 positive trials showed that although the magnitude of benefit declines as time from symptom onset to groin puncture increases, the treatment benefit also remains beyond 6 h after stroke onset, but it becomes nonsignificant after 7.3 h^8^.

More recent trials demonstrated that the time window for endovascular treatment can be extended up to 16^9^ or 24 h^10^ from stroke onset. As a result, current guidelines^11, 12^ recommend thrombectomy in the 6- to 24-h time window for patients meeting the DEFUSE-3 trial^9^ (CT or MRI perfusion with infarct volume mismatch) and DAWN^10^ (DWI or CTP assessment with clinical mismatch) criteria. These two randomized controlled trials (RCTs) revealed that EVT plus standard medical therapy resulted in better functional outcomes, when compared to standard medical therapy. Therefore, the decline in treatment effect appears to be strongly influenced by patient selection, and the concept of faster reperfusion is associated with better outcomes after the EVT has been challenged. These RCTs provide significant guidance in clinical practice for treating acute ischemic stroke patients.

However, these findings may not be fully generalizable to clinical practice due to the strict inclusion and exclusion criteria. Specifically, most trials selected patients based on additional imaging criteria, such as Alberta Stroke Program Early Computed Tomography Score (ASPECTS) thresholds, perfusion imaging and collateral status. For instance, in the DEFUSE 3 study, the estimated volume of the ischemic core and penumbral regions from the CT or MRI scans were calculated by RAPID software. However, this is not available in most hospitals in China due to the huge cost of the RAPID software. In clinical practice in Guangdong district in China, EVT patients are selected mainly based on the NIHSS score, onset time, ASPECTS, and conventional CT or MR mismatch without calculated. Whether this selection criteria will benefit patients is unclear, especially for patients beyond 6 h. Therefore, conducting a real world study in Guangdong district in China would be more meaningful.

The aim of the present study was to assess the clinical outcome of endovascular treatments performed for patients within and beyond 6 h without perfusion software in Guangdong district.

## Methods

Consecutive ischemic stroke patients within 24 h from 6 comprehensive stroke centers in Guangdong district (The First Affiliated Hospital of Jinan university, Maoming People’s Hospital, Shunde Hospital of Southern Medical University, Zhongshan People’s Hospital, Yunfu People’s Hospital and Affiliated Hospital of Guangdong Medical University) were enrolled into between March 2017 and May 2018. All eligible patients met the following inclusion criteria: (1) patients ≥ 18 years old; (2) patients who received emergency EVT within 24 h; (3) patients or their guardians who provided a signed informed consent. Patients with intracranial hemorrhage and refused to participate in this study were excluded. Neurointerventionist and neurologist selected patients for EVT mainly based on the NIHSS score, ASPECTS within 6 h and CT or MR perfusion mismatch by visual inspection beyond 6 h. The present study was registered in the Chinese Clinical Trial Registry (No. ChiCTR-OOC-17013052), and approved by the Medical Ethics Committee of the Host Unit of the First Affiliated Hospital of Jinan University. A total of 303 acute ischemic stroke patients with EVT from 6 comprehensive stroke centers were enrolled in Guangdong district. In this subgroup study, we included all patients who had acute proximal large vessel occlusion in the anterior circulation.

The demographic, clinical and neuroimaging data were collected from each center. Various data were included: age, gender, body mass index (BMI), vascular risk factors (hypertension, hyperlipidemia, diabetes and smoking habits), medical history (coronary heart disease, atrial fibrillation, valvulopathy, peripheral vascular disease, history of stroke, or TIA), the determination of whether oral antiplatelet drugs or anticoagulants were taken before stroke, blood glucose level, systolic and diastolic blood pressure, total cholesterol level, low-density lipoprotein cholesterol (LDL-C), the determination of whether intravenous thrombolysis was given, stroke etiology classification, site of occlusion, the anesthesia method, the time from onset to puncture, the time from puncture to reperfusion, NIHSS scores (at baseline, at 24 h and at seven days after EVT, and at discharge), mRS scores (pre-stroke, at discharge and at 90 days), baseline ASPECTS, modified TICI score of the main responsible artery before and after EVT, symptomatic intracranial hemorrhage (SICH) at 24 h, mortality, and the determination of whether stroke recurred at 90 days.

All radiologic data (ASPECTS and mTICI) were reviewed in a blinded fashion by two interventionists. A third experienced interventionist was consulted when there was any disagreement.

The etiology of stroke was divided into 3 categories (large-artery atherosclerosis, cardioembolism, and others/undetermined), based on the TOAST classification^13^. The occlusion site of the vessel was divided as follows: ICA, MCA M1, proximal MCA M2, and proximal ACA. The reperfusion status before and after the procedure was regarded as the modified TICI scale: 0, 1, 2a, 2b and 3^14^. Successful reperfusion was defined as a score on the mTICI scale ≥ 2b. Intracranial hemorrhage (ICH) at 24 h after treatment was classified as asymptomatic ICH and SICH, according to the Heidelberg bleeding classification^15^.

The efficacy outcome was favorable outcome (defined as a score on the modified Rankin scale of 0–2) at day 90. The score was assessed in person, or by telephone if an in-person visit was not feasible. The primary safety outcomes were death within 90 days and the occurrence of symptomatic intracranial hemorrhage within 24 h, defined as an increase of at least 4 points in the NIHSS score that was associated with brain hemorrhage on imaging within 24 h after symptom onset.

Quantitative variables were presented as mean ± standard deviation (SD) or median (interquartile range, IQR), as appropriate. Qualitative variables were presented in number and percentage. A multivariable logistic regression analysis to adjust for sex, age, BMI, vascular risk factors, medical history, dyslipidemia, NIHSS score and ASPECTS at baseline, the site of occlusion and anesthesia method, was also run to compare outcome between the two group. The favorable outcome was further evaluated with ordinal logistic regression, taking the whole range of mRS into account as a dependent variable adjusted for the above variables. Comparisons between the two groups for quantitative/ordinal variables were performed by Student’s *t*-test, Mann–Whitney U-test, or analysis of variance, as appropriate. Qualitative variables were compared by Pearson’s *X*^*2*^ or Fisher exact test, as appropriate. Significance was set at *P* < 0.05, and the *P*-values were two-sided. The statistical analysis was performed using SPSS Statistics 25.0 (IBM, Chicago, IL, USA).

### Ethical approval

All procedures performed in this study involving human participants were in accordance with the ethical standards of the national research committee and with the 1964 Helsinki declaration and its later amendments or comparable ethical standards.

## Results

Between March 2017 and May 2018, a total of 303 acute ischemic stroke patients with EVT were enrolled and 205 patients were proximal large vessel occlusion in the anterior circulation. Among these 205 patients, 13 (6.3%) patients were lost to follow-up at 90 days. Hence, a total of 192 patients were finally included. The median age of eligible patients was 66 (IQR, 59–76), and 60.9% (117/192) of these patients were male. Furthermore, among these patients, 125 (65.1%) patients received EVT within 6 h after onset were assigned to group A, while 67 (34.9%) patients received treatment at 6–24 h were assigned to group B. Median age was 67 (IQR, 59–76) in group A and 65 (IQR, 56–75) in group B. The most frequent site of occlusion was the middle cerebral artery M1 (47.2%, group A) and proximal M2 (53.7%, group B) (Table 1). The median onset-to-groin puncture time was 249 min (IQR, 189–303) in group A and 472 min (IQR, 412–615) in group B. The proportion of successful reperfusion in group A and B was 86.9% and 85.1%.

**Table 1:** Baseline characteristics of patients and features of EVT.

Characteristic	All (n = 192)	Group A (*n* = 125)	Group B (n = 67)	P (A vs. B)
**General data**				
Age (IQR), years	66 (59–76)	67 (59–76)	65 (56–75)	0.570
Male gender, no. (%)	117 (60.9)	70 (56)	47 (70.1)	0.064
BMI (IQR)	22.49 (20.28–24.97)	23.31 (20.57–25.28)	21.91 (20.02–24.44)	0.052
**Medical history, no. (%)**				
Smoking	54 (28.1)	36 (28.8)	18 (26.9)	0.824
Hypertension	93 (48.4)	58 (46.4)	35 (52.2)	0.442
Diabetes	37 (19.3)	23 (18.4)	14 (20.9)	0.660
Atrial fibrillation	46 (24.0)	32 (25.6)	14 (20.9)	0.450
coronary heart disease	21 (10.9)	14 (11.2)	7 (10.4)	0.874
Valvulopathy	24 (12.5)	19 (15.2)	5 (7.5)	0.123
Previous stroke or TIA	37 (19.3)	24 (19.2)	13 (19.4)	0.973
**Clinical data**				
Admission systolic blood pressure (mmHg)	144.5 (128.0–162.0)	146.0 (130.0–160.0)	141.0 (124.0–165.0)	0.333
Admission FBG (mmol/L) (IQR)	6.82 (5.54–8.95)	7.18 (5.71–9.10)	6.34 (5.27–8.67)	0.220
Admission LDL-C (mmol/L), (IQR)	3.02 (2.37–3.84)	3.02 (2.28–3.82)	3.05 (2.40–3.88)	0.864
Baseline NIHSS score (IQR)	14 (9–19)	16 (11–20)	12 (8–18)	0.009
Baseline ASPECTS (IQR)	10 (8–10)	10 (8–10)	9 (8–10)	0.034
**Stroke onset witnessed, no. (%)**				0.000
Yes	166 (86.5)	117 (93.6)	49 (73.1)	
No	26 (13.5)	8 (6.4)	18 (26.9)	
Symptoms were present on awakening	15 (7.8)	1 (0.8)	14 (20.9)	
Symptoms began during wakefulness	11 (5.7)	7 (5.6)	4 (6.0)	
**TOAST classification, no. (%)**				0.093
LAA	100 (52.1)	58 (46.4)	42 (62.7)	
CE	83 (43.2)	60 (48.0)	23 (34.3)	
Others/unknown	9 (4.7)	7 (5.6)	2 (3.0)	
**Occlusion vessels, no. (%)**				0.117
ICA	2 (1.0)	1 (0.8)	1 (1.5)	
M1	83 (43.2)	59 (47.2)	24 (35.8)	
Proximal M2	92 (47.9)	56 (44.8)	36 (53.7)	
Proximal ACA	15 (7.8)	9 (7.2)	6 (9.0)	
**Treatments, no. (%)**				
IVT + EVT, no. (%)	66 (34.4)	60 (48.0)	6 (9.0)	0.000
Direct EVT	126 (65.6)	65 (52.0)	61 (91.0)	0.000
**Anesthesia methods, no. (%)**				0.001
General anesthesia	27 (14.3)	26 (21.3)	1 (1.5)	
Local anesthesia	73 (38.6)	44 (36.1)	29 (43.3)	
Local anesthesia + conscious sedation	89 (47.1)	52 (42.6)	37 (55.2)	
mTICI score of the main responsible artery before EVT	0 (0–1)	0 (0–1)	0 (0–0)	0.950
mTICI score of the main responsible artery after EVT	3 (2b-3)	3 (2b-3)	3 (3–3)	0.128
Time from puncture to reperfusion (IQR)	50 (29–75)	50 (29–72)	50 (28–77)	0.648
NIHSS at 24 h	12 (5–19)	13 (5–21)	8 (4–16)	0.723
NIHSS at 7 days	10 (4–17)	10 (4–19)	8 (3–15)	0.823
NIHSS at discharge	6 (2–15)	8 (2–17)	4 (2–11)	0.766

Three data missing in group A of anesthesia method.

*IQR* interquartile range, *BMI* body mass index, *FBG* fasting blood-glucose, *LDL-C* low density lipoprotein cholesterin, *TIA* transient ischemic attack, *NIHSS* National Institutes of Health Stroke Scale score, *mRS* modified Rankin Scale, *ASPECTS* Alberta Stroke Program Early CT Score, *LAA* large artery atherosclerosis, *CE* cardiogenic, *ICA* internal carotid artery, *M1* the first section of medial cerebral artery, *M2* the second section of medial cerebral artery, *ACA* anterior cerebral artery, *IVT* intravenous thrombolysis, *EVT* endovascular treatment.

The NIHSS score at baseline (median 16, IQR12-20, group A; median 12, IQR8-18, group B; *P*-value = 0.009) and at 24 h (median 13, IQR5-21, group A; median 8, IQR4-16, group B; *P*-value = 0.046), and ASPECTS (median 10, IQR8-10, group A; median 9, IQR8-10, group B; *P* = 0.034) were higher in group A and the difference was statistically significant. Furthermore, the proportion of intravenous thrombolysis (48.0% vs. 9.0%, *P* = 0.000) and the anesthesia method (*P* = 0.001) also had a significant difference between the two groups. Anesthesia methods used in these two groups were general anesthesia (21.3% vs. 1.5%), local anesthesia (36.1% vs. 43.3%), and local anesthesia + conscious sedation (42.6% vs. 55.2%). In addition, more stroke onsets were witnessed in group A (93.6% vs. 73.1%). (Table 1).

The time from puncture to reperfusion (minutes) was similar (median 50, IQR29-72, group A; median50, IQR28-77, group B; *P* = 0.853). In addition, modified TICI score of the main responsible artery before (*P* = 0.950) and after EVT (*P* = 0.128), the NIHSS scores at seven days (*P* = 0.228) and at discharge (*P* = 0.129), and mRS scores at pre-stroke (*P* = 0.962) were not statistically different between the two groups. The other baseline characteristics were comparable across these two groups, but there was no statistical difference.

**Figure 1:**
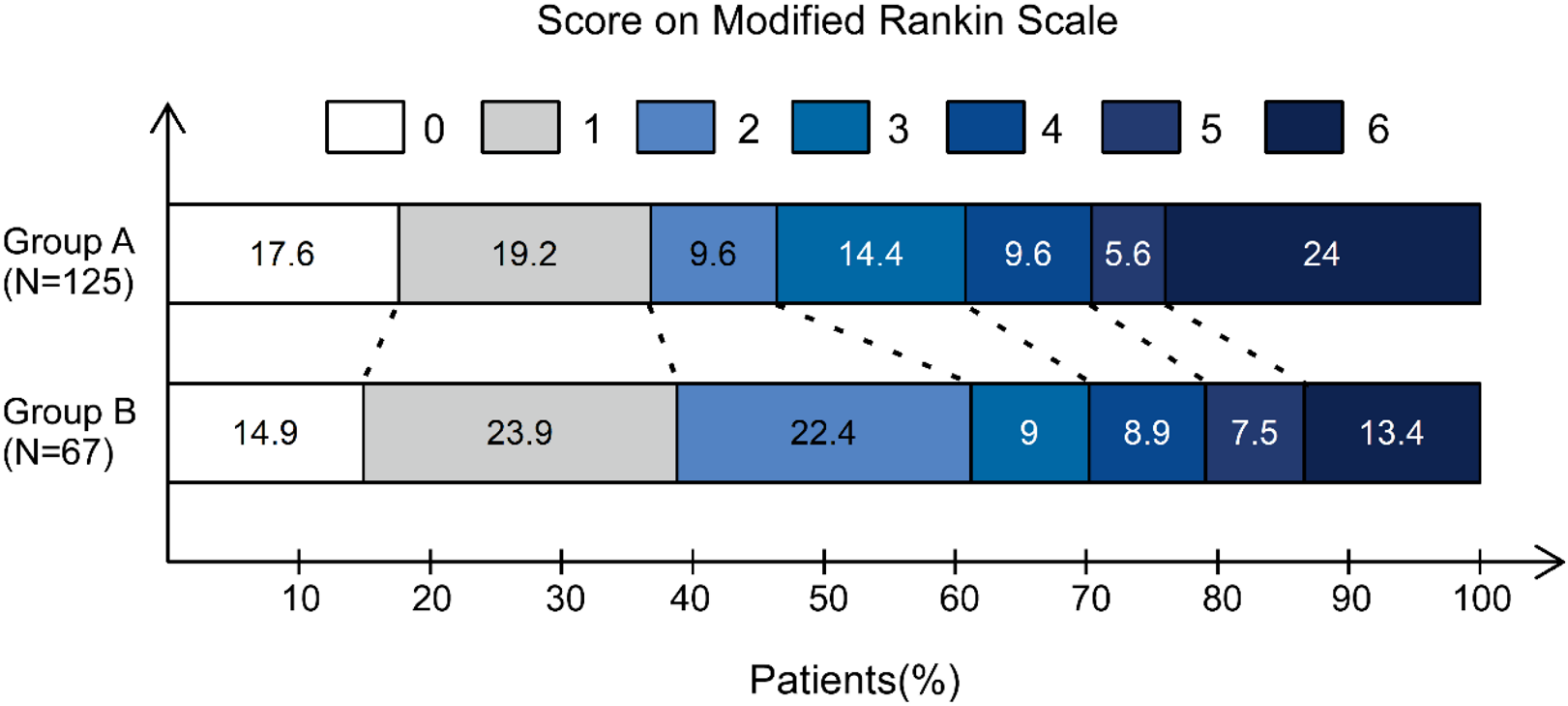
The modified Rankin scale scores at 90 days.

mRS scores at discharge (*P* = 0.310) and at 90 days (*P* = 0.258) were not statistically different between the two groups. As presented in Table 2 and the Fig. 1, 58 patients in group A and 41 patients in group B had a favorable outcome (mRS 0–2). The percentage of favorable outcome was 46.4% in group A vs. 61.2% in group B (*P* = 0.051). Patients treated within 6 h did not have substantial differences in clinical outcome, when compared with patients treated beyond 6 h.

**Table 2:** Clinical outcomes.

Outcome	All (*n* = 192)	Group A (*n* = 125)	Group B (*n* = 67)	*P* (A vs. B)
**Clinical outcome, no. (%)**				
mRS 0–2 at 90 days	99 (51.6)	58 (46.4)	41 (61.2)	0.051
**Safety outcome, no. (%)**				
Death at 90 days	39 (20.3)	30(24.0)	9 (13.4)	0.083
SICH at 24 h	22(11.5)	16 (12.8)	6 (9.0)	0.425

*SICH* symptomatic intracranial hemorrhage, favorable outcome, mRS 0–2.

The number of SICH cases within 24 h was 16 in group A and 6 in group B (Table 2), but there was no significant difference between these two groups (group A, 12.8%; group B, 9.0%; *P* = 0.425). The difference in rate of death in these two groups was not statistically significant (*P* = 0.083). A total of 30 (24.0%) deaths occurred within 90 days in group A. Among these cases, 22 cases were correlated to stroke, one case was correlated to pulmonary disease, and seven cases were correlated to other diseases, excluding pulmonary and cardiac disease. A total of 9 (13.4%) deaths occurred within three months in group B. Among these cases, seven cases were correlated to stroke, while two case were correlated to pulmonary disease.

## Discussion

Mortality and SICH and 90-day favorable outcome of patients in both two groups were not significantly different in the present real world study. These results suggesting that the prolonged time window for patients undergoing EVT also present with favorable and safety clinical outcome when compared to patients treated within 6 h. Traditionally, the extended time from onset to vascular recanalization is associated with bad functional outcome and higher intracranial hemorrhagic risk^16, 17^. In the past few years, shorter time from stroke onset to the start of EVT has been suggested as one of the important factors in the success of positive trials^18^. MR CLEAN Registry study have revealed that every hour of delay in time from onset to the start of EVT resulted in a 5.3% decrease in probability of functional independence^19^. However, our results seemed to be different from the above perceptions of “time is brain”. In fact, this study was under the premise of a 24-h time window, which was based on the DEFUSE and DAWN study. In this study, the favorable outcome of group B may be related to the following 3 factors. (1) Baseline NIHSS scores and ASPECTS of group B was lower than group A. This result indicates that group B may had smaller infarcts. (2) Most group B patients presented just over the 6 h time window, making it a comparable group from the start. In group B, 32.8% patients whose onset to groin puncture time were 6–7 h, 22.4% were 7–8 h, which account for more than half of the patients. (3) The selection of patients in group B mainly relied on CT or MR perfusion mismatch by visual inspection, which may lead to patients with more potential to benefit were being actively enrolled while patients with little benefit were excluded. However, all patients in group A were enrolled more actively.

The favorable outcome proportion in group A at 90 days was 46.4% in our study, which was similar with the Italian real world study (46.6%)^20^. And the proportion was 61.2% in group B, which was consistent with the result of observational study on EVT beyond 6 h (62%) by Alsahli^21^. However, the result of group B was above the Italian and other endovascular treatment real world studies^20, 22^. NIHSS scores at baseline in group B were lower than the Italian and other real world studies might be the main reason. In addition, visual assessment on CT or MR perfusion mismatch may cause bias towards benefit. In conclusion, time remains a key variable in predicting clinical outcome on a population basis, the very good results in the group B do not mean that we can wait. Because the selection of patients in group B mainly depends on perfusion, while perfusion maps are highly dependent on time and collateral flow. The favorable outcome just shows that for patients beyond 6 h, some of them can still benefit through selection in the real world in Guangdong district.

Among significant factors between group A and B, merely the determination of whether intravenous thrombolysis was given and the anesthesia method could be selected by the neurological physician, interventionist and anesthetist. The proportion of intravenous thrombolysis in group B was definitely low due to the intravenous thrombolysis time window (within 4.5 h). Therefore, the anesthesia method may be the minority selectable key factor that could affect the clinical outcome in the present real world study.

In the present real world data, general anesthesia was significant higher in group A (21.3% vs. 1.5%, group B). Through data analysis, we found that general anesthesia was used for severe patients with vomiting and extreme restlessness in group A, which may lead to deviations in results. Therefore, it may be the severity of stroke lead to the relatively poor clinical outcome of group A rather than the direct cause of general anesthesia in this real world. Though results of cohort study and systematic literature review also revealed that general anesthesia appeared to be associated with adverse clinical outcome and increased case-fatality after EVT^23, 24^. However it cannot be concluded that general anesthesia was associated with poor outcome in our study, because anesthesia methods were not associated with 90 days favorable or poor outcome through logistics regression. More evidence and big sample were needed to confirm the correlation between anesthesia methods and clinical outcome.

Low ASPECTS rating on non-contrast CT has been associated with poor outcome after reperfusion in EVT^25^. Inoue et al*.* suggested DWI-ASPECTS < 5 as the threshold of unfavorable prognosis for patients receiving EVT^26^, and the findings of the post-hoc analysis of the PROACT II trial revealed the interaction of baseline ASPECTS (> 7 vs. ≤ 7) with the intra-arterial treatment effect^27^. Therefore, non-contrast ASPECTS was highly predictive of outcome in previous studies^28, 29^. In the present study, the IQR of the ASPECT score was within 8–10 in both groups, indicating that both groups had small ischemic core volume. Although the median ASPECT score was higher in group A than in group B, the difference in IQR was not statistically significant. Hence, this may not have a significant effect to clinical outcome at 90 days.

Although the present study is a multicenter and prospective registration study conducted in China, the small number of stroke centers and little sample size, especially the EVT patients in group B, limited its statistical strength. A certain software is needed to calculate the CT perfusion or MRI diffusion-perfusion mismatch to improve the accuracy of the data. Therefore, a large sample size with more stroke centers and more advanced technology are needed to verify the difference between EVT within 6 h and 6–24 h.

In conclusion, for patients with acute ischemic stroke of proximal large vessel occlusion in the anterior circulation, there is no difference in clinical outcome between EVT within 6 h and 6–24 h from symptom onset to groin puncture in Guangdong district, China.
